# A Rare Case of Co-existing Non-small Cell Lung Carcinoma and Non-tuberculous Mycobacteria

**DOI:** 10.7759/cureus.50456

**Published:** 2023-12-13

**Authors:** Simran Agarwal, Girija Nair, Shahid M Patel, Priya Deshpande, Nikhil Sarangdhar

**Affiliations:** 1 Pulmonary Medicine, D. Y. Patil Medical College, Navi Mumbai, IND; 2 Pulmonary Medicine, D. Y. Patil Hospital, Navi Mumbai, IND

**Keywords:** non-tuberculous mycobacterial infection, non-tuberculous mycobacteria, non-small cell lung carcinoma, immunosuppression, immune exhaustion, rounded pneumonia, non-small cell lung cancer

## Abstract

A solitary pulmonary mass is commonly associated with malignancy; however, the possibility of co-existence with a pulmonary infection is rarely considered. Here, we present an extraordinary case, underscoring the importance of considering the possibility of concurrent lung cancer even when a bronchoscopy examination and bronchial lavage yield a positive mycobacterium culture result.

## Introduction

We report an unusual case of an elderly male with acute presentation and bronchial washings culture positive for non-tubercular mycobacterium.

## Case presentation

A 62-year-old male farmer presented with a low-grade fever of short duration, i.e., five days, a non-productive cough for five days, a loss of appetite for four days, and right-sided dull aching chest pain for one week. He denied contact with any known case of tuberculosis (TB), prior history of antitubercular drug intake, or any other chronic disease. There was no significant family history of respiratory disease or malignancy. The patient is a chronic bidi smoker, with a history of smoking up to five bidis per day for about 40 years.

**Figure 1:**
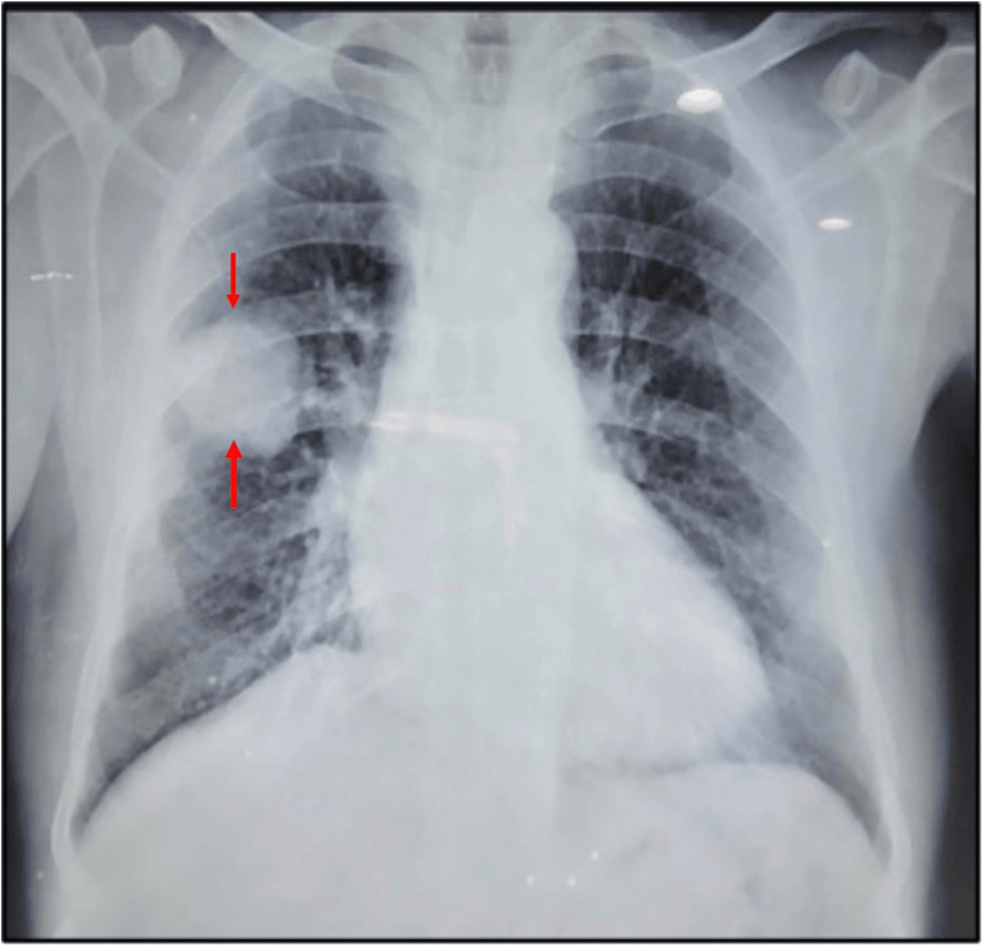
Chest radiograph posteroanterior view

On physical examination, his pulse rate, respiratory rate, blood pressure, and oxygen saturation were within normal limits, and the respiratory system examination was unremarkable. The routine serum and urinary investigations, including the haemogram and electrocardiogram, were all within normal limits. The patient’s chest radiograph (posteroanterior view) film revealed a right mid-zone, rounded, well-defined, radio-opaque lesion without calcification (Figure 1).

**Figure 2:**
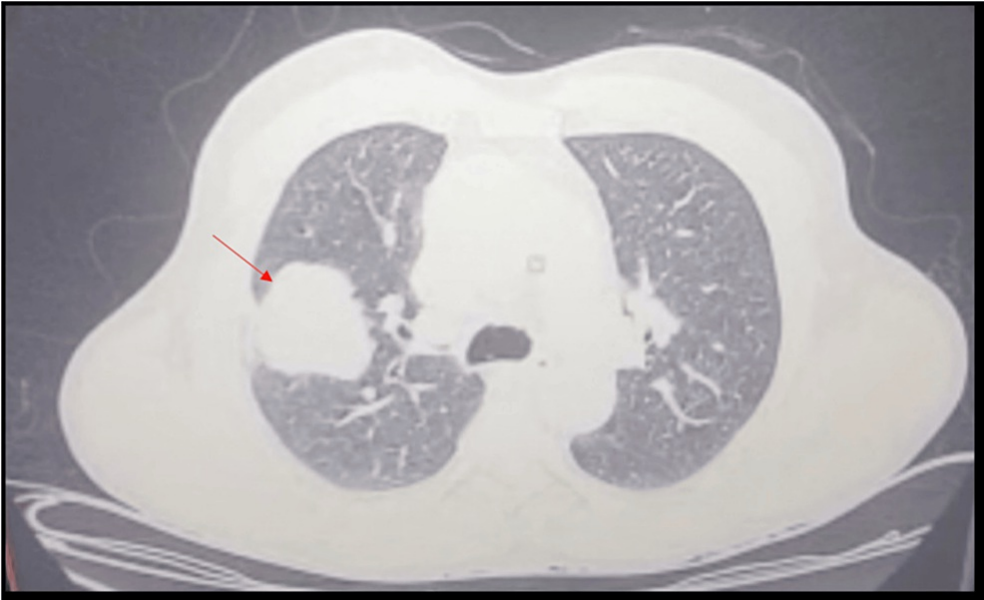
Chest HRCT (lung window)

**Figure 3:**
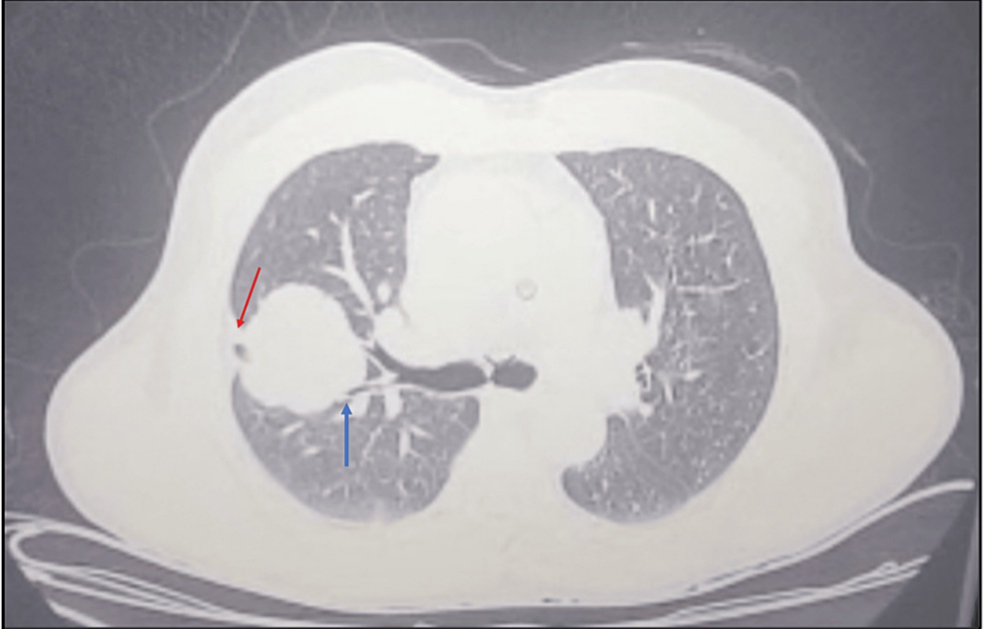
Chest HRCT (lung window) showing bronchus cut-off sign

**Figure 4:**
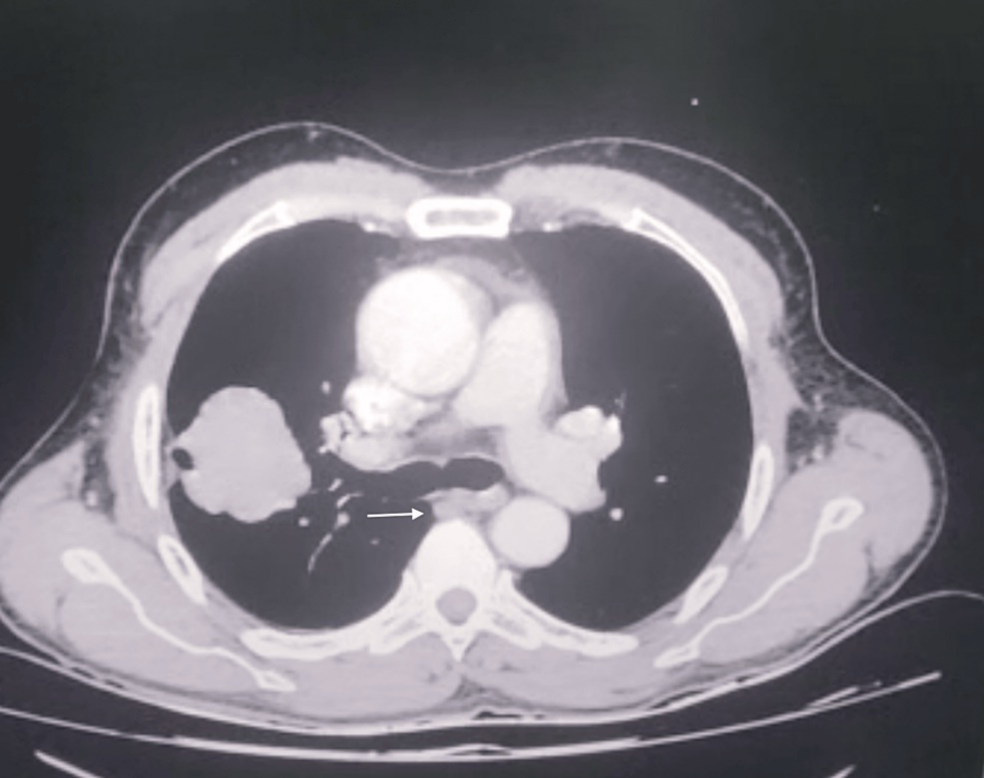
Chest HRCT (mediastinal window)

Expectoration smear acid-fast bacilli (AFB) microscopy and cartridge-based nucleic acid amplification test (CBNAAT) for *Mycobacterium tuberculosis* were negative. High-resolution computerised tomography (HRCT) of the chest showed a soft tissue density lesion measuring 4.0 x 3.8 cm (Figure 2) with micro-lobulations and a smooth margin, with an abrupt bronchus cut-off in the anterior segment of the right upper lobe and extending up to the right major fissure, leading to its retraction (Figure 3); furthermore, suspicious thickening was observed along the posterior aspect of this fissure. A few peri-fissural nodules along the right major and minor fissures in the right middle lobe were also observed. A few sub-centimetric, non-necrotic lymph nodes were present (Figure 4).

**Figure 5:**
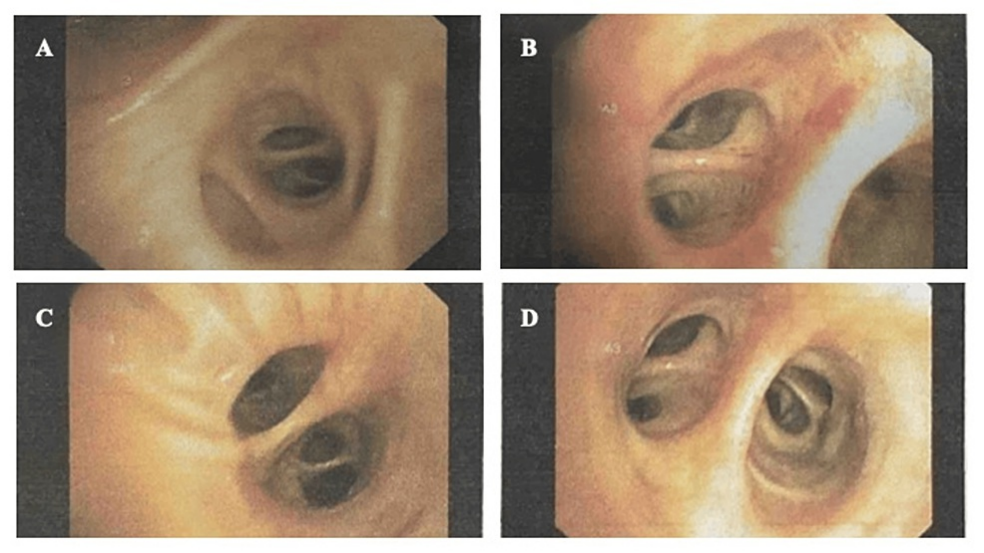
(A-D) Bronchoscopy with BAL images

A CT-guided-guided was advised; however, consent could not be obtained from the patient to undergo the procedure at the time. Bronchoscopy with bronchoalveolar lavage (BAL) was performed. No endobronchial lesion or mass was visualised during the procedure (Figure 5A-5D).

Ziehl-Neelsen staining showed AFB with 1+ grading in BAL fluid. *Mycobacterium tuberculosis* was not detected in CBNAAT of BAL fluid, whereas liquid culture (MGIT 960 system) confirmed the presence of mycobacteria other than tuberculosis (MOTT). Bronchial washing cytology was negative for malignant cells. Speciation of the MOTT could not be performed due to technical issues.

Based on the diagnostic assessment, a diagnosis of a solitary pulmonary nodule of non-tubercular mycobacterial origin was established. The patient was started on anti-tubercular treatment for drug-sensitive TB (isoniazid, rifampicin, pyrazinamide, and ethambutol - HRZE) under the National Tuberculosis Elimination Programme (NTEP) initially when a BAL smear was AFB positive. Following treatment initiation, the patient experienced near-complete resolution of fever, cough, and chest pain within three weeks; however, a chest radiograph on follow-up showed no reduction in the size of the nodule.

The patient and family counselled again, and a CT-guided lung biopsy of the right upper lobe mass was performed two months after starting anti-TB treatment. The tissue histopathology report revealed solid, non-small cell carcinoma composed of sheets of tumour cells possessing enlarged hyperchromatic, pleomorphic nuclei, and scanty cytoplasm. Immunohistochemistry was positive for cytokeratin (CK), epithelial membrane antigen (EMA), C-kit diffusely, and CK7 (very focally), with weakly expressed Pax-8 and a Ki-67 proliferation index of approximately 65%. The report was suggestive of high-grade solid non-small cell carcinoma (NSCLC).

## Discussion

Our patient presented with an acute history and a well-defined, rounded lesion in the right middle zone on a chest X-ray. Consent for a CT-guided biopsy had been denied initially, and bronchoscopic washings demonstrated AFB, which, on liquid culture with MGIT 960, revealed the presence of MOTT, or non-tubercular mycobacteria (NTM). A CT-guided done later was suggestive of NSCLC.

Radiologically, NTM pulmonary diseases are categorised as fibrocavitatory or nodular bronchiectatic diseases [1]. Our patient had a large, well-defined, rounded opacity, which required an in-depth assessment. The significance of this case report lies in highlighting that a positive culture result for sputum or bronchial lavage fluid indicative of NTM infection does not necessarily rule out the coexistence of lung cancer [2]. Even in situations where no malignant cells are observed in bronchial washings, as seen in this particular patient, further evaluation was warranted before lung cancer could be entirely excluded.

NTM and lung cancer

Hong et al. studied cases where NTM pulmonary disease manifested as a solitary mass resembling lung cancer [2]. They observed that these lesions typically displayed weak contrast enhancement (75%) and internal calcification (43%), which are more commonly seen in benign conditions like NTM pulmonary disease. Nevertheless, they also noted that certain CT features of solitary lesions, such as a lobulated border (71%), or pleural retraction (28%), overlapped with those seen in primary lung cancer.

While the link between NTM pulmonary disease and lung cancer is not widely recognised, chronic lung inflammation triggered by NTM is associated with an elevated risk of developing lung cancer [3,4]. Of the various types of NSCLC, prior studies have reported a higher incidence of the squamous cell carcinoma subtype [3,5].

In a retrospective matched cohort study of 252 patients, Liao et al. demonstrated a worse two-year survival rate in lung cancer patients on chemotherapy with a co-existing NTM infection, with bacterial pneumonia as the leading cause of death in this group [6]. He attributed these findings to two potential causes. One could be due to the persistent airway inflammation and epithelial damage from NTM, resulting in mucosal scarring and improper mucus secretion, which would lead to increased susceptibility to pneumonia, or it could be due to structural alteration of the lung, undermining the local immune response.

Immune exhaustion

Immune exhaustion (IE) is a condition associated with chronic infections and cancers, characterised by the inability of antigen-specific T-cells to eliminate the cognate antigen [7]. In their paper in 2021, Lombardi et al. described IE, a peculiar evolutionary characteristic of the immune system against chronic infections, and its contribution to the pathophysiology of NTM. Various studies have corroborated the involvement of CD4+ T-cells and lower levels of IFN-γ in patients with NTM lung disease (NTM-LD), particularly MAC [8-10].

CD4+ T-lymphocytes play an important role in generating an immune response against tumour cells through increased recruitment of CD8 T-lymphocytes [11,12]. They also directly attack the neoplastic cells through their action on NK cells and macrophages and activation of innate immunity [13]. Consequently, in chronic lung diseases, IE could repress an adequate immune response to malignant cells, leading to poor survival.

In general, a substantial body of evidence underscores the presence of IE in individuals with NTM-LD, along with the potential of PD-1 signalling inhibition to amplify IFN-γ production by cytotoxic T-lymphocytes, especially in MAC infections. The application of checkpoint inhibitors could be a promising and innovative therapeutic strategy, especially when combined with concurrent antimycobacterial treatment.

Management

Management of MOTT infections involves a multidisciplinary approach, including a combination of antimicrobial therapy, optimal treatment of underlying comorbidities, and surgical intervention on a case-to-case basis [14]. In our case, the patient was initially started on a standardised anti-TB regimen for drug-sensitive TB (HRZE) as per NTEP guidelines, to which he showed a favourable clinical response.

Distinguishing between NTM-LD and the progression of lung cancer is challenging due to the similar symptoms and radiological features they share. It is important to consider tubercular and non-tubercular mycobacterial infections as possible aetiologies for solitary pulmonary masses, particularly in regions with high TB prevalence. Therefore, to achieve a more accurate diagnosis, specific procedures like a transbronchial lung biopsy, bronchial brushing, bronchial lavage during bronchoscopy, or a CT-guided biopsy are essential. These procedures provide valuable diagnostic information that aids in distinguishing between the two conditions and ensuring appropriate management for the patient.

Bacteriological tests to identify pulmonary NTM in lung cancer patients prior to commencing chemotherapy may be performed. For patients with lung cancer undergoing chemotherapy, the presence of pulmonary NTM should heighten clinicians' suspicion regarding the presence of other bacterial lung infections. Comprehensive research is required to delve into the connection between the different subtypes of lung cancer and NTM infection. Further exploration into the clinical significance of pulmonary NTM in individuals with lung cancer is also required.

## Conclusions

This case emphasises the importance of considering the co-existence of an underlying malignancy, even in culture-positive pulmonary NTM, for solitary pulmonary nodules. There could be a cause-and-effect relationship between pulmonary NTM and lung malignancy. Chronic NTM could lead to malignancy development in a patient with risk factors, or immunosuppression in lung malignancy could lead to secondary infection with NTM. Awareness of this entity will help in early diagnosis and improve patient outcomes.
